# Occupational Exposure to Pesticides and Chronic Lymphocytic Leukaemia in the MCC-Spain Study

**DOI:** 10.3390/ijerph17145174

**Published:** 2020-07-17

**Authors:** Yolanda Benavente, Laura Costas, Marta Maria Rodríguez-Suarez, Juan Alguacil, Miguel Santibáñez, Javier Vila, Claudia Robles, Esther Alonso, Esmeralda de la Banda, Eva Gonzalez-Barca, Trinidad Dierssen-Sotos, Eva Gimeno Vazquez, Marta Aymerich, Elias Campo, José J. Jiménez-Moleón, Rafael Marcos-Gragera, Gemma Castaño-Vinyals, Nuria Aragonés, Marina Pollan, Silvia de Sanjose, Manolis Kogevinas, Adonina Tardón, Delphine Casabonne

**Affiliations:** 1 Cancer Epidemiology Research Programme, Institut Català d’Oncologia (IDIBELL), L’Hospitalet de Llobregat, 08908 Barcelona, Spain; lcostas@iconcologia.net (L.C.); crobles@iconcologia.net (C.R.); desanjose.silvia@gmail.com (S.d.S.); dcasabonne@iconcologia.net (D.C.); 2 Centro de Investigación Biomédica en Red: Epidemiología y Salud Pública (CIBERESP), 28001 Madrid, Spain; alguacil@dbasp.uhu.es (J.A.); J.Vila@epa.ie (J.V.); trinidad.dierssen@unican.es (T.D.-S.); jjmoleon@ugr.es (J.J.J.-M.); rmarcos@iconcologia.net (R.M.-G.); gemma.castano@isglobal.org (G.C.-V.); nuria.aragones@salud.madrid.org (N.A.); mpollan@isciii.es (M.P.); manolis.kogevinas@isglobal.org (M.K.); atardon@uniovi.es (A.T.); 3 Public Health Department, University of Oviedo, 33011 Oviedo, Spain; mrstsf@gmail.com; 4 Occupational Risk Prevention Service, Hospital Central University of Asturias, Public Health Service of the Principado of Asturias (SESPA), 33006 Oviedo, Spain; 5 Centro de Investigación en Recursos Naturales, Salud y Medio Ambiente (RENSMA), Universidad de Huelva, 21071 Huelva, Spain; 6 Faculty of Nursing, University of Cantabria—IDIVAL, 39008 Santander, Spain; miguel.santibanez@unican.es; 7 ISGlobal, 08903 Barcelona, Spain; 8 Departament de Ciències Experimentals i de la Salut, Universitat Pompeu Fabra (UPF), 08002 Barcelona, Spain; 9 Department of Pathology, Hospital Universitari de Bellvitge, L’Hospitalet de Llobregat, 08908 Barcelona, Spain; ealonso@bellvitgehospital.cat (E.A.); edlbanda@iconcologia.net (E.d.l.B.); 10 Hematology, Institut Català d’ Oncologia (IDIBELL), Universitat de Barcelona, L’ Hospitalet de Llobregat, 08908 Barcelona, Spain; e.gonzalez@iconcologia.net; 11 Faculty of Medicine, University of Cantabria—IDIVAL, 39011 Santander, Spain; 12 Hematology, Hospital del Mar, 08003 Barcelona, Spain; 94015@parcdesalutmar.cat; 13 Hematopathology Unit/Hemato-Oncology Department, Instituto de Investigaciones Biomédicas August Pi i Sunyer (IDIBAPS), 08001 Barcelona, Spain; aymerich@clinic.cat (M.A.); ECAMPO@clinic.cat (E.C.); 14 Centro de Investigación Biomédica en Red de Cáncer (CIBERONC), 28001 Madrid, Spain; 15 Department of Preventive Medicine and Public Health, University of Granada, 18071 Granada, Spain; 16 Instituto de Investigación Biosanitaria de Granada, Servicio Andaluz de Salud, Universidad de Granada, 18012 Granada, Spain; 17 Epidemiology Unit and Girona Cancer Registry, Oncology Coordination Plan, Department of Health, Autonomous Government of Catalonia, Catalan Institute of Oncology, Girona Biomedical Research Institute (IdiBGi), 17007 Girona, Spain; 18 Hospital del Mar Medical Research Institute (IMIM), 08001 Barcelona, Spain; 19 Department of Health of Madrid, Epidemiology Section, Public Health Division, 28035 Madrid, Spain; 20 National Center for Epidemiology, Carlos III Institute of Health, 28001 Madrid, Spain; 21 Instituto de Investigación Sanitaria (IIS) de Hierro, 28220 Majadahonda, Spain; 22 Sexual and Reproductive Health, PATH, Seattle, WA 98121, USA; 23 IUOPA Medicine Department, University of Oviedo, 33003 Oviedo, Spain; 24 Institute of Health Research of the Principality of Asturias-Foundation for Biosanitary Research of Asturias (ISPA-FINBA), 33003 Oviedo, Spain

**Keywords:** pesticides, job-exposure matrix, chronic lymphocytic leukemia, occupational exposure

## Abstract

We aimed to study the association between occupational exposure to pesticides and chronic lymphocytic leukemia (CLL) in Spain. Occupational exposure to pesticides (four insecticides, four herbicides and two fungicides) was evaluated using a job-exposure matrix for the Spanish population (MatEmESp) among 302 CLL cases and 1567 population controls in five regions of Spain, 2010–2013. Cumulative exposure scores (CES) were obtained by summing across the exposed jobs the product of prevalence, intensity and duration of exposure to each active substance. Principal components analysis (PCA) and logistic regression models adjusted for age, sex, region, education and occupational exposure to solvents were used. Around 20% of controls and 29% of cases were exposed to one or more pesticides. Compared to non-exposed, subjects in the highest tertile (3rd tertile) of CES of insecticides, herbicides, fungicides were more likely to have CLL [OR (95% CI), P-trend; 2.10 (1.38; 3.19), 0.002; 1.77 (1.12; 2.80), 0.12; and 1.67 (1.06; 2.64), 0.10, respectively). Following PCA, the first component (PC1, explaining 70% of the variation) equally led by seven active substances (the insecticide pyrethrin, all herbicides, all fungicides) was associated with a 26% higher odds of having CLL for 1-standard deviation increase in PC1 (95% CI: 1.14 to 1.40). These results confirm previous associations between CLL and exposure to pesticides and provide additional evidence by application groups and active substance. However, more research is needed to disentangle independent effects of individual active substances.

## 1. Introduction

Chronic lymphocytic leukemia (CLL) is a mature B-cell lymphoid neoplasm characterized by the presence of a clonal population of CD5+, CD19+ and CD23+ B-cell lymphocytes accumulating in the peripheral blood, bone marrow, lymph nodes and/or spleen [1]. CLL is the most common leukemia among the adult population with an annual European age-standardized incidence rate estimated around 3.79 per 100,000 person-years in 2000–2002 [2]. Its incidence is higher in males than in females in the Caucasian population and increases with age, with a median age at diagnosis of around 72 years [3]. CLL etiology is poorly understood although a pooled analysis of 15,186 controls and 2440 CLL cases from 13 case-control studies participating in the InterLymph consortium provided some insight. Here, significant inverse associations were observed with history of atopic conditions, of blood transfusion, and recreational sun exposure and moderate inverse association with smoking habit, while positive associations were found with height, hepatitis C virus seropositivity, having worked on a farm or as a hairdresser, and family history of hematological malignancies [4].

The elevated risks of CLL observed among farm workers are hypothesized to be related to exposure to several chemicals, such as pesticides, or/and biological agents. Potential carcinogenic mechanisms for pesticides exposure include oxidative stress, disruption of methyltransferases activity, endocrine disruption, DNA methylation and other epigenetic alterations, as well as reduction of S-adenosyl-methionine among others [5]. The International Agency for Research on Cancer (IARC) has classified the organochlorine lindane as carcinogenic to humans for haematological cancers (Group 1) while other pesticides examined, such as the herbicide glyphosate, the two organophosphate insecticides malathion and diazinon, as well as the organochlorine DDT were classified as probably carcinogenic to humans (Group 2A) while, the herbicide 2,4-D was considered as possibly carcinogenic to humans (Group 2B) [6]. Pesticides have been previously linked to CLL, although with inconsistencies. Among those examining CLL or lymphoid leukemias, some studies [7,8,9,10] but not all [11,12,13] observed positive associations with use of pesticides. However, there is little evidence available regarding is which specific active substances contribute to these observations.

We undertook the present population-based case-control analysis to evaluate the association between the exposure to specific pesticides (application groups and active substances) and CLL using a job exposure matrix for Spanish population (MatEmEsp) in the multi-case control study MCC-Spain.

## 2. Methods

### 2.1. Study Participants

Cases were recruited within the MCC-Spain study in collaboration with the International Cancer Genome Consortium on Chronic Lymphocytic Leukemia Project (ICGC-CLL, www.cllgenome.es and www.icgc.org). The main objective of the multicentric multi-case control study MCC-Spain was to investigate lifetime environmental, infectious, medical and occupational exposures, and genetic factors associated with five cancer types, including CLL [14]. In brief, the recruitment of both cases and controls were done simultaneously between March 2010 and July 2013. CLL cases were identified in 11 hospitals from five Spanish regions (Asturias, Barcelona, Cantabria, Girona and Granada), together with a set of frequency-matched controls by age, gender and region. Controls were randomly selected from lists of primary health care centres located within the catchment area of the hospitals recruiting CLL, contacted by phone on behalf of their general practitioner and invited to participate in the study. Subjects with communication difficulties or excess impairment of physical ability were excluded. Response rates were 87 and 53% for cases and controls, respectively. Information was requested through a computerized face-to-face interview performed by trained personnel (epidemiological questionnaire in Spanish is available at www.mccspain.org). The MCC-Spain study followed national and international directives on ethics and data protection (i.e., declaration of Helsinki and Spanish law on confidentiality of data, Ley Organica 15/1999 de 13 Diciembre de Proteccion de Datos de caracter personal-LOPD; approval number 2008/3121/I). All subjects were informed about the study and signed an informed consent form for study recruitment, collection and testing of biological samples and verification of clinical information. Finally, 302 CLL cases and 1567 controls with occupational information were included in this analysis (Supplementary Figure S1).

### 2.2. Outcome Definition

CLL cases were diagnosed according to the criteria of the International Workshop on Chronic Lymphocytic Leukaemia [1]. CLL and small lymphocytic lymphoma are considered the same underlying disease [15]. All diagnoses were morphologically and immunologically confirmed using flow cytometry immunophenotype and complete blood count. Given the generally indolent course of CLL, with most of the patients having a slow progression disease and with no treatment need, cases were defined as patients with CLL recruited within three years from diagnosis to interview. Newly diagnosed patients during the study period were considered as incident cases, and prevalent, otherwise.

### 2.3. Data Collection and Occupational Exposure Assessment

Self-reported lifetime occupational history was collected for all jobs held for more than one year. For each job, detailed information on job title, main activities or tasks performed, age at start and end, shift type, and average daily working hours were collected. Two industrial hygienists independently coded each job according to the Spanish National Classification of Occupations (CNO-94), which is the Spanish version of the International Standard Classification of Occupations 1988 (ISCO-88). The coding process was carried out blinded to the case-control status of the participants. When discrepancies occurred between the two coders (20% of job titles), an agreement was reached by consensus. Workplace exposures were then evaluated through a job exposure matrix designed for Spanish working conditions (MatEmESp) covering the period 1996–2005. MatEmEsp (www.matemesp.org) is a Spanish job-exposure matrix (JEM) developed in 2009 which includes occupational exposure estimates in five categories (hygiene, safety, ergonomics, psychosocial factors, and work conditions) based on job titles coded according to the CNO-94 [16] and includes quantitative indicators of probability and intensity (mg/m^3^) of exposure. Identification of occupations exposed to pesticides in MatEmESp was based on those considered in FINJEM [17] and was extensively extended and adapted to Spanish working conditions by local experts. Among 44 job titles exposed to pesticides according to MatEmEsp, we identified 27 in MCC-Spain study (Supplementary Table S1).

For pesticides, MatEmEsp selected active substances based on use, toxicological relevance, legal status of the use in Spain, and existence of professional exposure limits. Finally, ten different active substances in three application groups were considered: four insecticides (endosulfan, methomyl, pyrethrin, and chlorpyrifos), four herbicides (2,4D, atrazine, diquat, and diuron), and two fungicides (captan and thiram) [18]. For each job, an active substance specific exposure score was calculated as the result of the product of probability, intensity and duration (in years) of exposure to each specific active substance. Duration of exposure was computed considering the difference between age at last and first exposure. Since thiram was introduced in Spain in 1942, after applying MatEmEsp to MCC-Spain data, the first reported year of use was 1934. Consequently, for this active substance the period of exposure between 1934 and 1942 was excluded from the calculation of duration of exposure. Besides, when a participant reported more than one occupation exposed to an individual active substance during the same period of time, the duration was inversely weighted by the number of jobs during this overlapping period of time. Finally, Cumulative Exposure Scores (CES) were calculated for each participant by summing across the exposed jobs based on individual exposure scores for all pesticides, the pesticide application groups and individual active substances.

### 2.4. Statistical Analysis

Differences in the distribution of potential risk factors between cases and controls were assessed by means of chi-squared test for categorical variables, ANOVA and Wilcoxon rank-sum test for normal and non-normal continuous variables, respectively. Pearson’s correlation was used to assess the linear association between CES for pesticide application groups and individual active substances; Kendall rank correlation coefficient was chosen to explore the ordinal relation between duration, prevalence and intensity of exposure by application groups. Due to high correlation among individual active substances, principal component analysis (PCA) on the normalized CES for the ten individual active substances considered in MatEmESp was carried out. PCA is a reduction-dimension technique that using the covariance matrix aims to simplify a large set of variables into a smaller one that still contains most of the information in the large set. The obtained principal components are new uncorrelated variables that are constructed as linear combinations of the initial variables. The loadings can be interpreted as weights of each original variable to the new component. We estimated odds ratios (OR) and 95% confidence intervals (95% CI) associated with CLL through unconditional logistic regression models adjusting by age (<60, 60–70, and 71 or more), sex (male and female), region (Barcelona, Asturias, Girona, Cantabria and, Granada) and educational level (primary, secondary and, university). In the logistic regression models, the CES for the three pesticide application groups were included as categorical variable (i.e. one not exposed group and three exposed according to tertile exposure distribution in the exposed controls) and the principal components values (PC1, PC2 and PC3) as continuous (1-standard deviation (SD) increase). All models were also adjusted by exposure to solvents also using MatEmESp data. The potential effect of selected confounders (Supplementary Table S2) on the exposure estimates was also considered by including them in the model and assessing their significance using log-likelihood ratio tests and comparing the model with and without each of the confounders. When appropriate, a linear trend for exposed subjects was tested by fitting the model with the categorical variable as a continuous variable. Generalized additive models (GAM) were used to further explore the potential non-linear association between CLL and exposure to pesticides and each of the application groups. For each pesticide application group, mutual adjustments for the other pesticide groups were examined. Effect modification of confounders on the exposure was assessed using the log-likelihood ratio test, comparing the model with and without the interaction term. Sensitivity analyses involved: (a) excluding occupations with individual active substance prevalence of exposure below 25%; (b) considering as non-exposed those cases and controls who have never worked in any of the occupations identified by MatEmESP to be exposed to pesticides; and (c) stratifying the analyses by incident and prevalent cases. The significance level was established at 0.05 and all statistical tests were two-sided. Analyses and graphics were carried out using Stata Statistical Software: Release 14. College Station, TX, USA: StataCorp LP and R.3.6.0 [19].

## 3. Results

Cases were more likely to be male 67%, *p* = 0.02, to be recruited in the Barcelona area 69%, *p* < 0.0001, to have lower educational levels 75%, *p* = 0.08, to be older (mean age 65.9, *p* = 0.002), to report a family history of lymphoproliferative disorders 11%, *p* < 0.001 (Table 1) and to have higher median cumulative exposure scores for pesticides and pesticide application groups than controls (Supplementary Figure S2). Among controls, occupational exposure to pesticides was associated with male sex, older age, living in Barcelona, primary education, type II diabetes and high body mass index (Supplementary Table S2).

Cumulative exposure scores (CES) for pesticide application groups and individual active substances were highly correlated. Pearson correlation between CES for herbicides and fungicides was 1.00 and the correlations between individual herbicide and fungicide active substances and the insecticide pyrethrin were above 0.97. The lowest correlations were observed between the insecticide methomyl and the rest of individual active substances (Supplementary Figure S3a Duration of exposure to pesticides overall or by application groups was positively correlated with prevalence and intensity of exposure with Kendall’s tau correlation coefficient above 0.71 (Supplementary Figure S3b).

Overall, around 20% of controls and 29% of cases were exposed to pesticides. Of them, 106 (7%) controls and 42 (14%) cases were classified in the highest tertile (3rd tertile, t3) of the CES for pesticides, which was positively associated with CLL (OR = 1.60; 95% CI = 1.04 to 2.46; Table 2) although with no statistically significance trend (*p*-trend = 0.11). By pesticide application groups, those in the highest CES tertile were more likely to have CLL compared to the non-exposed subjects (OR_t3 vs unexposed_ = 2.10; 95% CI = 1.38 to 3.19; OR = 1.77; 95% CI = 1.12 to 2.80 and OR = 1.67; 95% CI = 1.06 to 2.64, for insecticide, herbicide, and fungicide, respectively), although a significant linear trend was only observed for insecticide (*P*-trend = 0.002).

**Figure 1:**
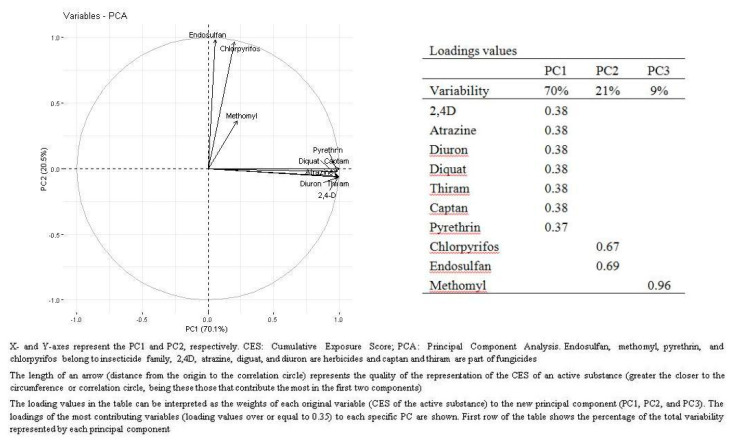
Loading plot and values of principal component analysis (PCA) of CES and pesticide chemical agents (active substances) in the MCC-Spain.

As shown by the PCA (Figure 1), the first principal component (PC1), which explained 70% of the variability of CES, was equally mainly loaded by the CES of each of the four herbicides (loadings for each active substance: 0.38) and the two fungicides (loading for each active substance: 0.38) as well as the pyrethrin insecticide (loading: 0.37), whereas PC2 (21% of variability explained) was mostly loaded by the CES of two insecticides: endosulfan (loading: 0.69) and chlorpyrifos (loading: 0.67). The third component (PC3) was mainly loaded by the insecticide methomyl (loading: 0.96). Participants working in farming as the longest occupation were best represented by PC1 whereas those working as cleaners as their longest activity were better grouped within PC3 (Supplementary Figure S4). Controlling for the other PCs, PC1 and PC3 were associated with CLL (OR_1-SD increase_ = 1.26; 95%CI = 1.14 to 1.40 and OR_1-SD increase_ =1.12; 95%CI = 1.00 to 1.26, for PC1 and PC3, respectively) whereas no associations were observed for PC2 (Table 3). According to the GAM results, there was some evidence for a non-linear dose response between the CES for insecticides, PC3 and CLL probability (*p* = 0.004 and *p* = 0.04 for insecticides and PC3, respectively) (Supplementary Figure S5).

Further adjustment of the CES or PC by potential confounders (smoking, type II diabetes, BMI ever work in agriculture/farming and, family history of LPD) did not have any impact on the exposure estimates (data not shown). No effect modifications were observed between confounders and exposures (data not shown). Sensitivity analyses, excluding occupations with prevalence of exposure of 25% or below from the analysis or considering as non-exposed subjects who did not report to have ever worked in any of the occupations exposed to pesticides in MatEmESp, did not materially change the results (data not shown). Stratifying cases by incident and prevalent CLL, we obtained higher estimates for incident CLL for ever exposed to any pesticides and the three pesticides application groups (OR = 1.63; 95% CI = 1.02 to 2.61, OR = 1.64; 95% CI = 1.03 to 2.63, OR = 1.93; 95% CI = 1.16 to 3.21, and OR = 1.77; 95% CI = 1.07 to 2.95, for pesticides, insecticides, herbicides and fungicides, respectively) compared to non-exposed and for 1-SD increase in PC1 (OR = 1.33; 95% CI = 1.16 to 1.53) (Supplementary Table S3).

## 4. Discussion

Applying MatEmESp, a Spanish specific JEM, to the MCC-Spain case-control study, we observed a positive association between CLL and the highest tertile (3rd tertile) of the cumulative exposure score (CES) to all pesticides and to the three pesticide application groups, after controlling for solvents. In particular, within the highly correlated data, PCA further identified two distinct pesticide patterns positively associated with CLL: (1) exposure to seven active substances (the insecticide pyrethrin, all herbicides, all fungicides) and (2) exposure to methomyl.

Of the four herbicides included in MatEmESp, 2,4D has been classified as possibly carcinogenic to humans (2B) in IARC monographs [6]. Unfortunately, using MaEmESp in the MCC-Spain study, it was not possible to disentangle the effect of the individual herbicides or fungicides active substances separately due to the high correlation between their CES. As part of the AGRICOH consortium, a pooled analyses of three large agricultural worker cohorts including 2430 non-Hodgkin lymphoid malignancies (NHL) cases diagnosed among 316,270 farmers, a recent study (2019) not included in the IARC monographs, assessed the association between 33 individual active substances from various pesticide application groups and NHL overall as well as by subtypes [20]. While this study has intrinsic limitations related to various questions of the study design, of the 33 examined active substances, only the insecticide deltamethrin was associated with CLL. Interestingly, deltamethrin is a pyrethroid or a synthetic version of pyrethrin, which is typically derived from *Chrysanthemum cinerariifolium* [21].

Another occupational study carried out in Italy [22] found that exposure to the fungicide captafol, chemically similar to captan, was significantly associated with non-Hodgkin lymphoma, while paraquat (a herbicide belonging to the same chemical family as diquat) was non significantly related to CLL. Although their results were based on a limited sample size, they are in accordance with our observations, especially considering that similar active substances were explored. Coggon et al. [23] found no significantly elevated risk or trends between background exposure for more than 1 year to phenoxy herbicides (which includes 2,4 D) and CLL. Insecticide exposure has been widely linked to CLL. In the large EpiLymph study, increased risks with occupational exposure to organic and inorganic pesticides were observed, with the highest associations amongst those ever exposed to organophosphate insecticides [8]. Similarly, in the Agricultural Health Study, a US-based prospective cohort of farmers and commercial pesticide applicators, associations with terbufos (an organophosphorous insecticide) and DDT (a chlorinated insecticide) were associated with CLL [7]. Furthermore, women participating in the Women’s Health Initiative in US who reported that a commercial service (ever) applied insecticides in their immediate surroundings had significant higher risk of CLL [10]. These results are in line with our results on methomyl, a common active substance in commercial fly bait that was highly correlated with the cleaning sector in our study. However, we found a null association we found between CLL and the second pesticide pattern obtained in PCA, defined by the other two active substances in the insecticide application group. However, results from the large prospective European cohort EPIC, did not provide evidence of an association for lymphoid leukemias when the ALOHA-JEM was applied to estimate exposure to pesticides, herbicides and insecticides [12]. Similarly, a case-control study in France based on 77 CLL cases revealed null results for occupational exposure to pesticides [11]. Subjects whose longest occupation was farming were well represented by the first pesticide pattern identified by our PCA that was characterized by exposure to seven active substances (the insecticide pyrethrin, all herbicides, all fungicides). These results confirm previous associations observed with farm work [3] and suggest that exposures to pesticides through other occupations are not a strong risk factor for CLL. However, we cannot rule out the possibility that other farm-related exposures, including biological agents and different lifestyles, were involved in the higher risk observed for farm work.

The main strengths of our study include the relatively large sample size that allowed us to analyze exposures using an updated and rigorous classification of CLL, as well as the use of a specific Spanish JEM. One of the main limitations of the present study is the use of a JEM to estimate pesticide exposure, since this can lead to exposure misclassification due to Berkson-type error [24,25]. JEMs represent a widely used tool for exposure assessment in occupational studies [26], they are cost-efficient [27] and perform better than self-reported occupational exposures in large population-based studies [28]. Nevertheless, the JEM used in this study (MatEmEsp) only refers to the 1996–2005 period while the use of pesticides in our population started earlier. Although the use of pesticides may have changed over time, we assumed that earlier exposures should have been at least as high as the ones estimated by MatEmEsp. Although this could be a relevant source of exposure misclassification, its potential effect is likely to be non-differential and, would result in the attenuation of estimates for binary and continuous exposure estimates [29], which may strengthen our conclusions. The fact of inclusion of prevalent cases might be a cause of concern since people who have died shortly after diagnosis could have had a different exposure to pesticides than those who survived. However, although survival bias cannot be discarded, our estimates were higher after restricting analyses to incident cases. The lower response rate of controls compared with cases in addition to their slightly younger age, and their higher educational level might have introduced selection biases. Finally, some of the reported associations may be due to chance because of the multiple comparisons performed, while independent effects were difficult to assess given the strong correlations between the active substances considered.

In conclusion, using exposure estimates from a JEM adapted to Spanish working conditions, we observed positive associations between higher occupational exposure to pesticides and CLL. In particular, despite highly correlated data, we identified combinations of application groups and specific chemical agents related to the disease. Further studies using an improved exposure assessment and including non-occupational exposures are warranted to unravel the role of specific active substances on CLL.

## Figures and Tables

**Table 1:** Characteristics of controls and chronic lymphocytic leukemia (CLL) cases.

	CONTROL n (%)	CLL n (%)	*p* ^1^
	*n* = 1567	*n* = 302	
Male	945 (60)	203 (67)	0.02
Age at interview (mean, sd)	63.6 (10.83)	65.9 (10.69)	0.002
Region: Barcelona	807 (51)	209 (69)	<0.0001
Education: Secondary or higher	477 (30)	74 (25)	0.08
Alcohol intake (gr/day): current (mean/sd)	12.05 (16.44)	9.55 (13.56)	0.11
Alcohol intake (gr/day): past (mean/sd)	20.34 (27.89)	23.02 (34.69)	0.84
Ever smoker	911 (58)	178 (59)	0.79
Type II diabetes	214 (14)	46 (15)	0.42
BMI (mean/sd) at interview	26.96 (4.35)	27.32 (4.13)	0.14
Height (cm) (mean/sd)	166.1 (8.32)	166.91 (8.42)	0.16
Ever work in agriculture/farming	312 (20)	103 (34)	<0.0001
Family history of LPD	81 (5)	32 (11)	<0.0001

BMI: Body mass index, sd: standard deviation, LPD: lymphoproliferative disorder, CES: cumulative exposure score. ^1^ *p* value for chi-squared test for categorical variables; ANOVA test for continuous variables if normality is satisfied, Wilcoxon rank-sum test otherwise. Exposure pesticides includes exposure to any of the insecticides, herbicides or fungicides considered in MatEmEsp. Insecticides include exposure to endosulfan, methomyl, pyrethrin, and chlorpyrifos, herbicides include exposure to 2,4D, atrazine, diquat, and diuron and fungicides include exposure to captan and thiram. Missing by variables in controls/cases, respectively: alcohol intake (14/31%), BMI (4/8%), height (3/8%), housewives (6/4%), family history of LDP (5/6%), education (0/2%); CES pesticides, insecticides, CES herbicides, and CES fungicides less than 1% in both cases and controls.

**Table 2:** Odds ratios (OR) and 95% confidence intervals (CI) of CLL and cumulative exposure score (CES): overall and by pesticide families.

	CONTROL n (%)	CLL n (%)	OR (95% CI) ^1^	OR (95% CI) ^2^
Unexposed to pesticides ^3^	1248 (80)	214 (71)	Ref	Ref
Ever exposed to pesticides	319 (20)	88 (29)	1.27 (0.93 to 1.73)	1.26 (0.92 to 1.73)
1st Tertile	105 (7)	18 (6)	0.93 (0.54 to 1.59)	0.94 (0.55 to 1.62)
2nd Tertile	106 (7)	27 (9)	1.19 (0.74 to 1.93)	1.20 (0.74 to 1.95)
3rd Tertile	106 (7)	42 (14)	**1.64 (1.07 to 2.51)**	**1.60 (1.04 to 2.46)**
*p*-value ^4^			0.085	0.105
Unexposed to insecticides ^5^	1249 (80)	214 (71)	Ref	Ref
Ever exposed to insecticides	318 (20)	88 (29)	1.28 (0.93 to 1.74)	1.27 (0.93 to 1.74)
1st Tertile	101 (6)	17 (6)	0.77 (0.43 to 1.35)	0.78 (0.44 to 1.37)
2nd Tertile	111 (7)	26 (9)	0.98 (0.61 to 1.60)	0.99 (0.61 to 1.60)
3rd Tertile	104 (7)	44 (15)	**2.13 (1.40 to 3.24)**	**2.10 (1.38 to 3.19)**
*p*-value ^4^			**0.001**	**0.002**
Unexposed to herbicides ^6^	1340 (86)	230 (76)	Ref	Ref
Ever exposed to herbicides	226 (14)	72 (24)	1.39 (0.99 to 1.96)	1.39 (0.99 to 1.95)
1st Tertile	75 (5)	17 (6)	1.13 (0.64 to 1.99)	1.15 (0.65 to 2.03)
2nd Tertile	66 (4)	17 (6)	1.03 (0.57 to 1.86)	1.03 (0.57 to 1.86)
3rd Tertile	84 (5)	36 (12)	**1.81 (1.14 to 2.85)**	**1.77 (1.12 to 2.80)**
*p*-value ^4^			0.11	0.12
Unexposed to fungicides ^7^	1322 (84)	229 (76)	Ref	Ref
Ever exposed to fungicides	245 (16)	73 (24)	1.27 (0.90 to 1.77)	1.26 (0.90 to 1.77)
1st Tertile	75 (5)	15 (5)	0.99 (0.55 to 1.80)	1.01 (0.55 to 1.83)
2nd Tertile	83 (5)	20 (7)	0.94 (0.54 to 1.63)	0.94 (0.54 to 1.63)
3rd Tertile	86 (5)	36 (12)	**1.71 (1.08 to 2.70)**	**1.67 (1.06 to 2.64)**
*p*-value ^4^			0.11	0.10

The CES tertiles were defined according to the tertile distribution in the exposed controls for each of the specific exposures with the third tertiles including the highest values. ^1^ Model adjusted by age, sex, region, and education. ^2^ Model adjusted by age, sex, region, education, and ever occupational exposed to solvents. ^3^ Exposure to pesticides includes exposure to any of the insecticides, herbicides or fungicides considered in MatEmEsp. CES tertile cut-offs (<41.82, 41.82 to 230.07, >=230.08). ^4^ *p*-value for linear trend. ^5^ Insecticides includes exposure to endosulfan, methomyl, pyrethrin, and chlorpyrifos. CES tertile cut-offs (<6.4, 6.52 to 10.56 to 16.33, >=16.34).^6^ Herbicides includes exposure to 2,4D, atrazine, diquat, and diuron. CES tertile cut-offs (<71.44, 71.44 to 250.03, >=250.04). ^7^ Fungicides includes exposure to captan and thiram. CES tertile cut-offs (<13.28, 13.28 to 46.47, >=46.48). In bold, *p*-value < 0.05.

**Table 3:** Odds ratios (OR) and 95% confidence intervals (CI) of CLL for 1-standard deviation increase in principal component.

PC ^1^	OR ^2^ (95% CI)	OR ^3^ (95% CI)	OR ^4^ (95% CI)
PC1	**1.27 (1.15 to 1.42)**	**1.27 (1.14 to 1.41)**	**1.26 (1.14 to 1.40)**
PC2	0.92 (0.77 to 1.11)	0.92 (0.77 to 1.10)	0.92 (0.74 to 1.13)
PC3	1.11 (0.99 to 1.24)	1.11 (0.99 to 1.24)	1.12 (1.00 to 1.26)

^1^ PC: principal component (PC1, highly loaded by herbicides and fungicides and the insecticides, explained 70% of the variability. PC2, highly loaded by two insecticides explained 21% of the variability. PC3, loaded by methomyl, explained 9% of the total variability. ^2^ Model adjusted by age, sex, region, and education. In bold, *p*-value < 0.05. ^3^ Model adjusted by age, sex, region, education, and ever occupational exposed to solvents. In bold, *p*-value < 0.05. ^4^ Model adjusted by age, sex, region, education, ever occupational exposed to solvents, and PC1, PC2 and PC3. In bold, *p*-value < 0.05.

## Supplementary Materials

The following are available online at https://www.mdpi.com/1660-4601/17/14/5174/s1, Figure S1: Flow chart of included subjects, Table S1: Occupations considered exposed to pesticides in MatEmEsp, Figure S2: Distribution of normalized CES by pesticides overall and functional groups according to participant status, Figure S3-a): Pearson correlation of CES of pesticides functional groups and pesticides chemical agents (active substances), Figure S4-b): Kendall correlation of exposure duration, prevalence and intensity and pesticides groups (I, H and F), Figure S5: Participants according to their coordinates of PC1 and PC3 stratified by their longest occupation exposed to pesticides, Figure S6: Generalized Additive Models for Cumulative Exposure Score (CES) to pesticides: overall, by pesticide families and principal components, Table S2; Characteristics of controls occupationally exposed to pesticides in MatEmEsp, Table S3: Odds Ratio (OR) and 95% confidence intervals (CI) of CLL and exposure to pesticides: overall, by pesticide families and 1-SD increase in principal components.
