# Supplementary material for: Occupational Exposure to Pesticides and Chronic Lymphocytic Leukaemia in the MCC-Spain Study
Source: Int J Environ Res Public Health. 2020 Jul 17;17(14):5174. doi: 10.3390/ijerph17145174 (PMC7400560; doi:10.3390/ijerph17145174)
Supplement: Supplementary file 1 [file ijerph-17-05174-s001.pdf]

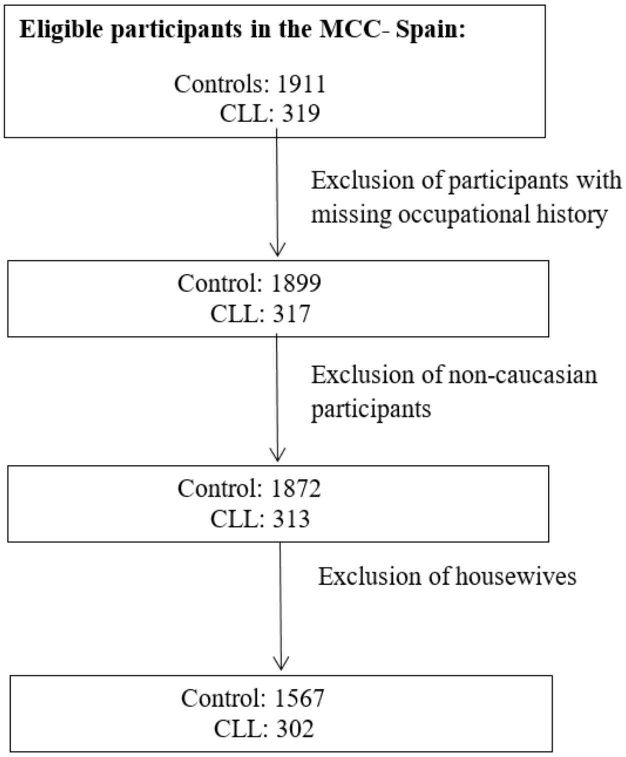

Figure S1. Flow chart of included subjects. CLL: Chronic Lymphocytic Leukemia

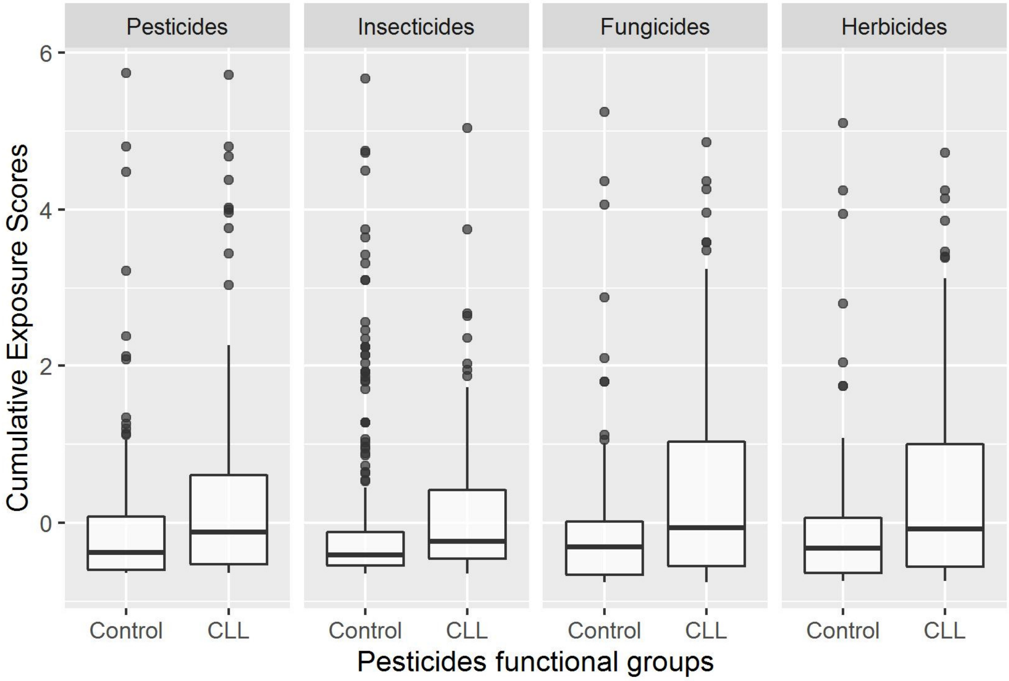

Figure S2. Distribution of normalized CES by pesticides overall and functional groups according to participant status. CES: Cumulative Exposures Scores, CLL: Chronic Lymphocytic Leukemia

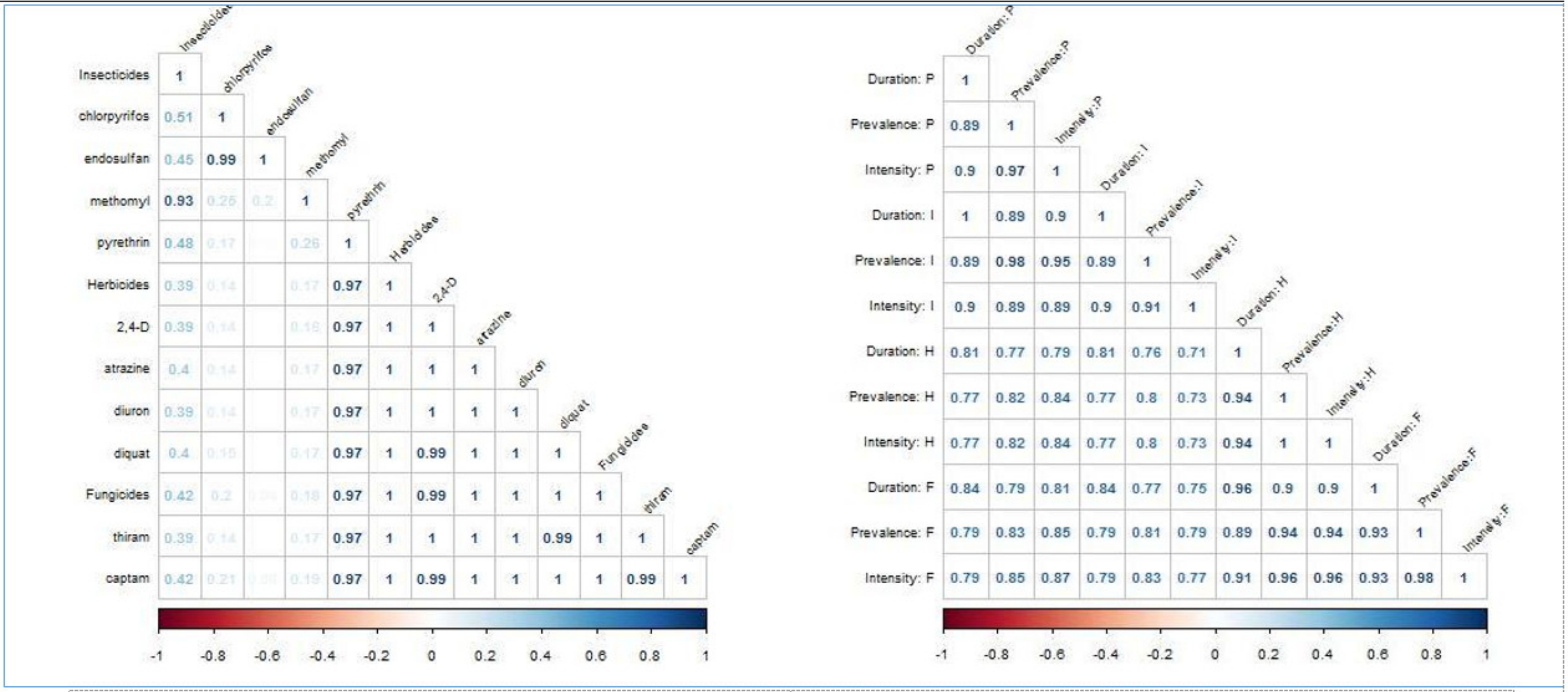

a) Pearson correlation of CES of pesticide functional groups and pesticide chemical agents (active substances)

b) Kendall correlation of exposure duration, prevalence and intensity and pesticide groups (I, H and F)

Figure S3. Pearson correlation of CES of pesticides functional groups and pesticides chemical agents (active substances). CES: Cumulative Exposure Score. P: pesticides, I: insecticides, H: herbicides, F: fungicides. Insecticides (I): endosulfan, methomyl, pyrethrin, and chlorpyrifos. Herbicides (H): 2,4D, atrazine, diquat, and diuron. Fungicides (F): captan and thiram.

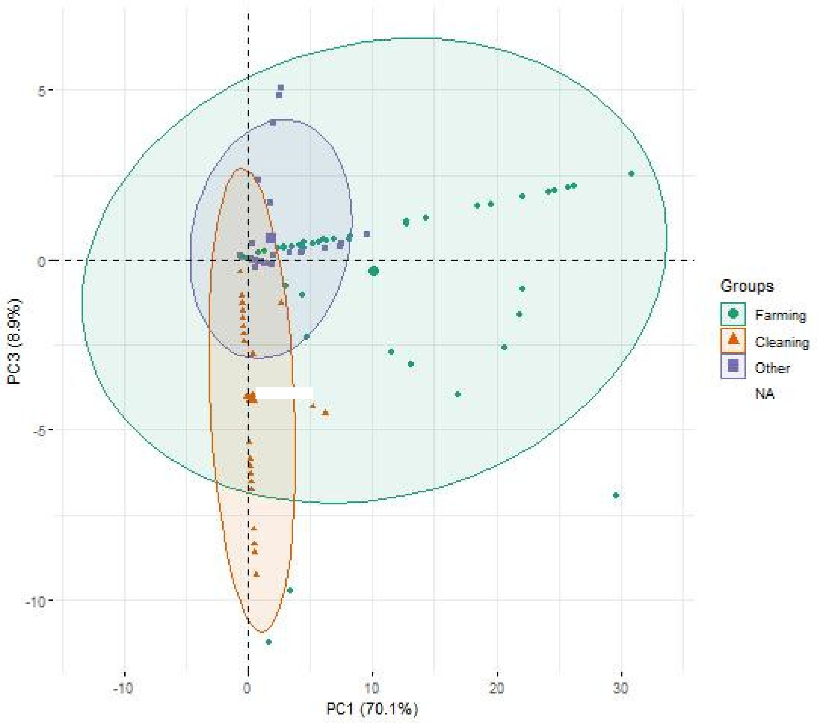

Figure 4. Participants according to their coordinates of PC1 and PC3 stratified by their longest occupation exposed to pesticides. Among those exposed to pesticides (n = 407), in 31% represented their longest occupation. By sector, 40% were farmers (n = 50), 33% cleaners (n = 42) and 27% were employed in activities related to transportation, wood industry or other sector (n = 34).

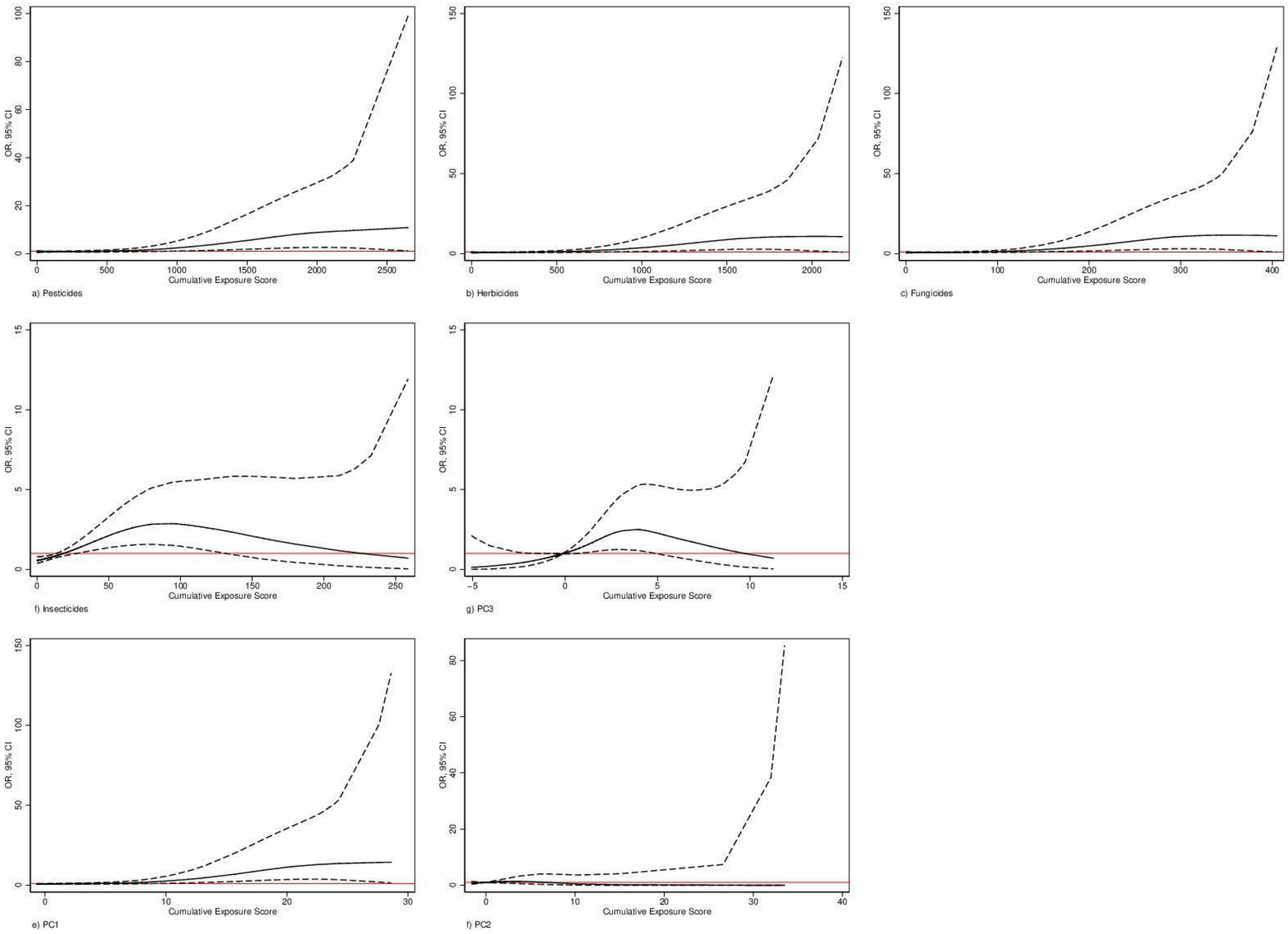

Figure S5. Generalized Additive Models for Cumulative Exposure Score (CES) to pesticides: overall, by pesticide families and principal components. OR: Odds Ratio, 95% CI: 95% Confidence Interval. Generalized lineal model with 3 knots adjusted by age, sex, region, education, exposure to solvents, and principal components mutually adjusted. Pesticides: nonlinearity P-value = 0.36; Insecticides: non-linearity P-value = 0.004; Herbicides: non-linearity P-value = 0.41; Fungicides: nonlinearity P-value = 0.35; PC1: nonlinearity P-value = 0.11;PC2: nonlinearity P-value = 0.14; PC3: nonlinearity P-value = 0.04. Note: the scales differ between the graphs.

Table S1. Occupations considered exposed to pesticides in MatEmESp.

CNO-94[1]	ISCO-88[2]	Description[3]	MCC-Spain[4]
1401	1311	General managers in agriculture, hunting, forestry/ and fishing	
2711	2211	Biologists, botanists, zoologists and related professionals	
2113	2213	Agronomists and related professionals	
2130	2223	Veterinarians	Yes
3112	3212	Agronomy and forestry technicians	Yes
3113	3213	Farming and forestry advisers	
3123	3222	Sanitarians	
3122	3227	Veterinary assistants	
6021	6111	Field crop and vegetable growers	
6012	6112	Tree and shrub crop growers	Yes
6111	6121	Dairy and livestock producers	Yes
6122	6122	Poultry producers	
6129	6129	Market-oriented animal producers and related workers not elsewhere classified	
6210	6130	Market-oriented crop and animal producers	
6241	6141	Forestry workers and loggers	Yes
6242	6142	Charcoal burners and related workers	Yes
7250	7143	Building structure cleaners	
7911	7421	Wood treaters	Yes
8040	814	Wood-processing- and papermaking-plant operators	Yes
8141	8141	Wood-processing-plant operators	Yes
8142	8142	Paper-pulp plant operators	Yes
8143	8143	Papermaking-plant operators	Yes
8050	815	Chemical-processing-plant operators	
8152	8152	Chemical-heat-treating-plant operators	
8159	8159	Chemical-processing-plant operators not elsewhere classified	Yes
8240	824	Wood-products machine operators	Yes
8340	8240	Wood-products machine operators	Yes
8530	8331	Motorised farm and forestry plant operators	Yes
9121	9132	Helpers and cleaners in offices, hotels and other establishments	Yes
9410	9211	Farm-hands and labourers	Yes
9440	9212	Forestry labourers	Yes
1701	1311	General managers in agriculture, hunting, forestry/ and fishing	
2712	2213	Agronomists and related professionals	
6011	6111	Field crop and vegetable growers	Yes
6022	6112	Tree and shrub crop growers	Yes
6121	6121	Dairy and livestock producers	Yes
6112	6122	Poultry producers	
6119	6129	Market-oriented animal producers and related workers not elsewhere classified	
6230	6130	Market-oriented crop and animal producers	
6220	6141	Forestry workers and loggers	
9420	9211	Farm-hands and labourers	Yes
9430	9211	Farm-hands and labourers	Yes
9700	932	Manufacturing labourers	Yes
9800	933	Transport labourers and freight handlers	Yes

[1] Spanish classification of occupations (1994).[2] International Standard Classification of Occupations (1988). [3] Description based on ISCO-88. [4] Job available in MCC-Spain.

Table S2. Characteristics of controls occupationally exposed to pesticide in MatEmESp.

	Exposed to pesticides		
	**Never n (%)** **n = 1248**	**Ever n (%)** **n = 319**	**P[1]**
Male	721 (58)	224 (70)	0.0001
Age at interview (mean, sd)	62.9 (11.0)	66.2 (9.9)	0.0001
Region: Barcelona	613 (49)	194 (61)	0.0002
Secondary or higher	721 (58)	54 (17)	<0.00001
Alcohol intake (gr/day): Current (mean/sd)	11.8 (16.0)	12.9 (18.3)	0.61
Alcohol intake (gr/day): Past (mean/sd)	19.3 (26.3)	24.7 (33.1)	0.05
Ever smoker	716 (57)	195 (61)	0.22
Diabetes Type II	153 (12)	61 (19)	0.001
BMI (mean/sd) at interview	26.8 (4.4)	27.7 (4.2)	0.0001
Height (mean/sd)	166.3 (8.4)	165.5 (8.0)	0.13
Family history of LPD	67 (5)	14 (4)	0.53

BMI: Body Mass Index, sd: standard deviation, LPD: Lymphoproliferative disorder. [1] chi-squared test for categorical variables; ANOVA test for continuous variables if normality is satisfied, Wilcoxon rank-sum test otherwise. Missing in alcohol intake (n = 179, 14%, and 48, 15%, in never and ever exposed to pesticides, respectively). Missing in BMI (n = 51, 4%, and N = 15, 5%, in never and ever exposed to pesticides, respectively). Missing in height (n = 41, 3%, and n = 12, 4% in never and ever exposed to pesticides, respectively).

Table S3. Odds ratio (OR) and 95% confidence intervals (CI) of CLL and exposure to pesticides: overall, by pesticide families and 1-SD increase in principal components.

	**CONTROL n (%)**	**Incident n (%)**	**OR[1] (95% CI)**	**Prevalent n (%)**	**OR[1] (95% CI)**
**Ever exposed to**					
Pesticides	319 (20)	36 (32)	1.63 (1.02 to 2.61)	52 (27)	1.07 (0.73 to 1.57)
Insecticides	318 (20)	36 (32)	1.64 (1.03 to 2.63)	52 (27)	1.07 (0.73 to 1.58)
Herbicides	226 (14)	30 (27)	1.93 (1.16 to 3.21)	42 (22)	1.14 (0.75 to 1.73)
Fungicides	245 (16)	30 (27)	1.77 (1.07 to 2.95)	43 (23)	1.03 (0.68 to 1.56)
PC1[1]			1.33 (1.16 to 1.53)		1.20 (1.06 to 1.37)
PC2[1]	1564	108	0.75 (0.32 to 1.78)	186	0.93 (0.75 to 1.15)
PC3[1]			1.19 (0.94 to 1.51)		1.12 (0.97 to 1.29)

1 Model exploring exposure to pesticides, insecticides, herbicides and fungicides adjusted by age, sex, region, education and ever occupational exposed to solvents. In bold, P-value< 0.05. Models for PC1, PC2 and PC3 adjusted by age, sex, region, education, ever occupational exposed to solvents and mutually adjusted bu other PC. In bold, P-value< 0.05. SD: Standard Deviation.
